# Regional Liver Function Assessment Using ^99m^Tc-GSA SPECT/CT Scintigraphy in Malignant Perihilar Biliary Tumor Undergoing Major Liver Resection: A Dual-Center Cohort Study

**DOI:** 10.1245/s10434-025-17207-x

**Published:** 2025-04-17

**Authors:** Ming Xiao, Jianping Song, Zhenlei Lyu, Xin Huang, Yuewei Zhang, Liang Wang, Zuoxiang He, Tianyu Ma, Can Li, Han Cao, Qijia Zhang, Canhong Xiang, Jiahong Dong

**Affiliations:** 1 Department of Hepatobiliary Surgery, The Second Hospital of Shandong University, Jinan, China; 2 Hepatopancreatobiliary Center, Beijing Tsinghua Changgung Hospital, Key Laboratory of Digital Intelligence Hepatology (Ministry of Education), School of Clinical Medicine, Tsinghua Medicine, Tsinghua University, Beijing, China; 3 Department of Engineering Physics, Tsinghua University, Beijing, China; 4 Department of Nuclear Medicine, Beijing Tsinghua Changgung Hospital, School of Clinical Medicine, Tsinghua Medicine, Tsinghua University, Beijing, China; 5 Department of Nuclear Medicine, The First Medical Centre, Chinese PLA General Hospital, Beijing, China; 6 Medical Data Science Center, Beijing Tsinghua Changgung Hospital, School of Clinical Medicine, Tsinghua Medicine, Tsinghua University, Beijing, China; 7 Research Unit of Precision Hepatobiliary Surgery Paradigm, Chinese Academy of Medical Sciences, Beijing, China

## Abstract

**Background:**

Cholestasis can lead to unreliable results of routine liver function assessment tests in clinical practice and the functional cutoff value of hepatectomy is still unclear. The aim of this study was to determine which ^99m^Tc-GSA scintigraphy functional indicators can predict post-hepatectomy liver failure (PHLF) in patients before major liver resection due to malignant perihilar biliary disease. In addition, it aimed to assess the efficiency of functional future liver remnant (FLR) assessment of ^99m^Tc-GSA scintigraphy indicators.

**Patients and Methods:**

A ^99m^Tc-GSA scintigraphy was performed prior to planned surgery in 187 patients, including 81 patients with major liver resection. The ^99m^Tc-GSA scintigraphy parameters including functional liver volume (FLV), ratio of the FLR functional volume to body weight (FLV_FLR_–BWR), and predictive residual index (PRI) were calculated from radioactive count in regions of FLR and total liver (TOTAL). Morphological liver volume (MLV) was calculated from computed tomography and standardized by standard liver volume (SLV). The efficacy of these parameters in predicting PHLF was compared using generalized linear mixed models and receiver operating characteristic (ROC) curve analysis.

**Results:**

PHLF occurred in 22 patients, who showed lower MLV_FLR_/SLV, FLV_FLR,_ FLV_FLR_/FLV_TOTAL,_ FLV_FLR_–BWR, and PRI and higher resection rate (*P* < 0.05 for all) than patients without PHLF. After adjusting for clinical parameters, a decreased FLV_FLR_–BWR (odds ratio, OR 0.17; 95% confidence intervals, CI 0.05–0.53) was found to be an independently significant indicator in the model of GLMM. FLV_FLR_–BWR (0.835) had the highest ROC among all liver functional indicators.

**Conclusions:**

The FLR functional parameter preoperatively estimated from preoperative ^99m^Tc-GSA scintigraphy protocol is a promising tool for regional liver function assessment, and it can distinguish high-risk patients who may develop PHLF with malignant perihilar biliary tumor undergoing major liver resection.

**Supplementary Information:**

The online version contains supplementary material available at 10.1245/s10434-025-17207-x.

Cholangiocarcinoma is a malignant tumor originating from epithelial cells in different parts of the bile duct. Perihilar cholangiocarcinoma (pCCA) is the most common type, representing more than 50% of cholangiocarcinoma cases.^1,2^ Owing to the pathological characteristics of its submucosal infiltrative growth and the biological behavior of easily invading surrounding vital blood vessels, a hemihepatectomy or extended hemihepatectomy combined with caudate lobe resection or even vascular resection and reconstruction is required to achieve the goal of radical tumor resection.^2,3^ However, these procedures are associated with high risk, especially extensive liver resection, leading to a high incidence of complications (18–77%) and mortality (1–11%) exceeding those of other hepatobiliary procedures.^2,4^ In addition, patients with pCCA often have obstructive jaundice, and bile stasis can lead to a significant decrease in liver synthesis and metabolism. The increase in bile salts inhibits the oxidation of hepatic cytochrome P450 and reduces aerobic metabolism, thereby increasing oxidative stress and hepatocyte apoptosis.^5^ Precisely assessing liver function of the future liver remnant (FLR) before surgery is crucial to reduce the incidence of postoperative complications and post-hepatectomy liver failure (PHLF).^6^

Current clinical preoperative liver assessments primarily include laboratory biochemical tests, Child–Pugh score, indocyanine green (ICG) clearance test, and computed tomography (CT) to evaluate the overall or regional hepatic function.^6–9^ Although biochemical tests such as transaminases and alkaline phosphatase reflect liver function to a certain extent, they fail to provide information about regional liver function and are highly susceptible to extrahepatic factors. The Child–Pugh score, initially developed to predict surgical risks in patients with cirrhosis undergoing shunt surgery for variceal bleeding, contains subjective assessment criteria and cannot differentiate which Class A patients are at risk of liver failure.^8^ In 2015, the ALBI grade was introduced to address the limitations of the Child–Pugh classification;^10^ however, there are limited data supporting its superiority in preoperative hepatic function assessment for patients with pCCA. The ICG clearance test, often used to evaluate whole liver function, cannot directly evaluate the function of FLR. Morphologic liver volume measured from computed tomography (CT) can be utilized for FLR regional liver assessment.^2,6–9,11^ However, this approach assumes uniform functionality across all liver segments.^11,12^ Patients with pCCA with bile obstruction exhibit a certain incidence of comorbidities such as fibrosis, cirrhosis, or steatosis.^13^ Furthermore, they may undergo procedures such as biliary drainage, portal vein embolization (PVE), hepatic vein embolization (HVE), or associating liver partition and portal vein ligation for staged hepatectomy (ALPPS) prior to hepatectomy. The extent of vascular invasion between liver lobes may also vary. Consequently, these factors contribute to heterogeneity in regional liver function, complicating the precise assessment of FLR. Morphologic volumes derived from CT scans may be misleading and not accurately reflect functional differences among different lobes. Effectively assessing FLR function in patients with pCCA remains a complex issue for surgeons.

The ^99m^Tc-galactosyl human serum albumin (^99m^Tc-GSA) scintigraphy is a nuclear imaging technique used to quantitatively assess regional liver function. ^99m^Tc-GSA binds specifically to the asialoglycoprotein receptor (ASGPR) on the hepatocyte membrane surface, a process unaffected by serum bilirubin level.^14,15^ Through appropriate modeling, indicators representing the number of functional hepatocytes can be calculated.^14^ This method has been validated for its usefulness in patients with bile duct cancer with obstructive jaundice and PVE, albeit with a limited sample size of bile duct cancer in single-center retrospective studies.^16–19^ However, the potential superiority of ^99m^Tc-GSA single-photon emission computed tomography (SPECT)/CT over CT and the ICG test in assessing FLR in patients with cholestasis, as well as the accuracy of its cutoff values for predicting PHLF in patients with pCCA undergoing major liver resection, remains uncertain. To date, there is no multicenter cohort study that has assessed FLR function in pCCA using ^99m^Tc-GSA SPECT/CT. This study aims to analyze the influence of functional FLR on PHLF using ^99m^Tc-GSA SPECT/CT and to establish a safe FLR cutoff value for major liver resection in patients with pCCA across two centers.

## Patients and Methods

### Patients

Consecutive patients were prospectively recruited at Beijing Tsinghua Changgung Hospital, and a prospectively maintained database was reviewed for all patients at Chinese People’s Liberation Army (PLA) General Hospital between January 2010 and September 2023. Inclusion criteria were as follow: patients aged ≥ 18 years with a diagnosis of malignant perihilar biliary tumor; patients completed ^99m^Tc-GSA SPECT/CT within 1 month prior to hepatectomy or 3 weeks after PVE; and patients who underwent R0 tumor resection by hepatectomy for more than or equal to three liver segments during hospitalization with regular postoperative follow-up. Exclusion criteria were as follows: elevated bilirubin level caused by nonbiliary obstructive factors; inadequate image quality of SPECT/CT; and a history of radiation therapy, chemotherapy, or immunotherapy before surgery. According to the distribution of CT, ICG, and ^99m^Tc-GSA SPECT/CT indicators between PHLF and non-PHLF groups,^11,20^ as well as the current PHLF group and non-PHLF group, *α* was set to 0.025 on one side, and statistical efficiency was calculated using PASS 2021 software. The statistical efficiency of all the above indicators was greater than 0.800 (the minimum is 0.844), and the sample size was sufficient. This study complies with the Declaration of Helsinki and began after the institutional review board approval at the Beijing Tsinghua Changgung Hospital (no. 20356-0-01; Chinese Clinical Trial Registry number: ChiCTR2000041507) and Chinese PLA General Hospital (no. S2014-052-01).

### *The *^*99m*^*Tc-GSA SPECT/CT Data*

A ^99m^Tc-GSA SPECT/CT was performed as described by prior research.^21,22^ Scans acquisition were accomplished using Discovery NM/CT 670 (General Electric Healthcare, USA) and Symbia T6 (Siemens Medical Solutions, Germany) at two centers. GSA (3 mg, Beijing Shihong Drug Research Center, Beijing, China) was labeled with ^99m^Tc (185 MBq, HTA Company Limited, Beijing, China) at least 10 min before injection. Patients assumed a supine position on the examination table, and a nonenhanced CT scan under standard conditions was performed first. Then, SPECT detectors were placed toward the patient’s liver and heart, and radioactivity was measured as soon as the intravenous bolus injection of ^99m^Tc-GSA was complete. The dual detectors continuously rotated through 180° at a speed of 1 min/rotation. A total of 26 frames with 128 × 128 matrixes were acquired. The first 25 frames took 1 min per frame and the last frame took 5 mins. The slice thickness for image reconstruction was 0.54 cm. The SPECT images and parallel CT images were fused in built-in software.

### *Future Liver Remnant Parameters from *^*99m*^*Tc-GSA SPECT/CT*

On the basis of the CT images, regions of interest (ROIs) including the whole liver and FLR were semiautomatically drawn by commercial software named IQQA^®^-3D (EDDA Technology, Shanghai, China), avoiding large vessels and the gallbladder. Resected liver regions were determined by two reviewers (J.P.S. and X.H.) according to the operation records and were checked by another two experienced hepatobiliary surgeons (L.W. and C.H.X.).

### Calculation of the Morphological Liver Volume

Morphological liver volume (MLV) was calculated by summation of the liver area on CT images by IQQA^®^-3D. FLR volume was standardized by the standard liver volume (SLV):^23^ $\text{SLV}=706.2\times \text{BSA}+2.4$, where body surface area (BSA) is determined by the patient’s weight and height. Resection rate was calculated by dividing the volume of the excised liver by the total liver volume (TLV).

### Calculation of the Functional Liver Volume

Functional liver volume (FLV) was calculated depending on the radioactivity of each voxel in ROIs.^24^ The maximum voxel count of the last frame was determined. The functional volume of each voxel with a count above 80% of the maximum voxel count was estimated to be 86.24 mm^3^. The functional volume of voxels with a count from 35 to 80% of the maximum voxel count was calculated on the basis of the voxel count. Voxels with a count below 35% were regarded as background. The FLV of the ROI is the sum of functional volume of all voxels in the region. According to the clinical value of the future liver remnant volume‐to‐body weight (FLRV/BW) on 348 patients with pCCA,^25^ the combination of live volume and body weight is a comparable risk prediction factor of PHLF. Referring to this parameter, a new predictive index of FLR liver function, ratio of the FLR functional volume to body weight (FLV_FLR_–BWR), was calculated preoperatively. FLV_FLR_–BWR (%) was calculated as FLV of FLR divided by body weight.

### Calculation of the Disappearance Rate Constant

The disappearance rate constant (*k*-value, GSAK) represents the ^99m^Tc-GSA absorption rate constant for hepatic cells.^24^ The *k*-value was calculated from the liver uptake curve using the clearance half-time: $C\left(t\right)={C}_{\text{max}}\left(1-{e}^{-kt}\right)$, where ${C}_{\text{max}}$ is the maximum count in ROIs.

### Calculation of the Predictive Residual Index

Predictive residual index (PRI), a useful parameter in preoperative prediction of PHLF, was calculated on the basis of the parameters of the *k*-value and FLV. The formula used to calculate the PRI was: $\text{PRI}= \sum \frac{{k}_{i} \times {\text{FLV}}_{i}}{{k}_{n} \times \text{Total FLV}}$, where *k*_*i*_ and FLV_*i*_ are the *k*-value and FLV of the *i*th ROI, respectively, *k*_*n*_ is the normal *k*-value calculated from normal volunteers,^24^ and total FLV is the preoperative FLV of the whole liver.

### Preoperative ICG Clearance Test

The indocyanine green retention rate at 15 min (ICGR15), evaluated within 1 week prior to hepatic resection, served as a referenced standard for routine clinical assessment of liver function. The clearance rate of indocyanine green (ICGK) was also calculated.^26^ The standardized preoperative assessment of remnant liver function in biliary cancer with or without PVE, as determined by the ICG clearance test, was quantified as ICGK-F by scholars in Japan.^20,26,27^

### Technique of PVE and Hepatectomy

On the basis of former research,^27,28^ PVE was performed when FLR was less than 40% of the total liver base on morphological volume or when the ICGK-F was below 0.05. A ^99m^Tc-GSA SPECT/CT was conducted at least 3 weeks after PVE and prior to the scheduled liver resection. In jaundiced patients, the intervention was carried out after the serum total bilirubin had decreased to at least less than 5 mg/dL following percutaneous transhepatic biliary drainage (PTBD). PVEs were performed by the ipsilateral approach, in which the portal vein was accessed by an ultrasound-guided puncture of the portal vein to be embolized, as previously reported.^27^ Patients with insufficient hypertrophy (FLR < 40% of total liver) underwent hepatic vein embolization (HVE) of the middle hepatic vein 3 weeks after PVE on the basis of the experience reported in a previous study.^29^

All hepatic resections were meticulously performed by the team of experienced surgeons (C.H.X. and J.H.D.) at two centers. The safe extent of hepatectomy was decided following the Chinese consensus decision tree.^7^ The demarcation of the resection was precisely delineated utilizing a combination of vascular staining, intraoperative ultrasonography, and anatomic benchmarks. The transection of liver parenchyma was executed using elementary surgical clamps, the Cavitron Ultrasonic Surgical Aspirator (CUSA, Tyco Healthcare, Mansfield, Massachusetts, USA), and electrocautery techniques. Intermittent inflow control via selective occlusion of the portal vein was implemented—clamping for a duration of 15 min followed by a release of 5 min—as necessitated by the surgical circumstances. Intraoperative parameters, including duration of the operation, volume of blood loss, and transfusion, were documented. Cholangioenterostomy and excision of caudate lobe were routinely performed in patients with preliminary diagnosis of pCCA. Vascular reconstruction included hepatic arterial and/or portal vein reconstruction.

### Outcome Measures

The main endpoint of this study was post-hepatectomy liver failure. Post-hepatectomy liver failure was defined, scored, and graded according to the International Study Group of Liver Surgery (ISGLS) criterion.^30^ A comprehensive physical assessment was conducted, and detailed clinical histories were gathered from all patients. Laboratory liver evaluations consisted of the quantification of serum concentrations of albumin, total bilirubin, alanine and aspartate aminotransferases, and international normalized ratio. Blood serum analyses were conducted preoperatively and on or subsequent to the first postoperative day.

### Statistical Analysis

Continuous variables are expressed as mean ± standard deviation (SD) or median (interquartile range, IQR), and were compared using the Student’s *t*-test or Wilcoxon rank sum test according to the data distribution. Categorical variables are presented as frequency (percentage) and were compared using the *χ*^2^ test or Fisher’s exact test.

Generalized linear regression models (GLMMs) were used to assess the associations between liver volume variables and PHLF, in which participants and research center were considered as the first and second levels, respectively. The univariate and multivariate analyses were all conducted. We adjusted for the demographic variables [i.e., age, gender, and body mass index (BMI)], preoperative variables (i.e., ALT, TBIL, ALB, INR, PTBD, and PVE), and operative variables (i.e., operation time and hemorrhage) in multivariate analyses. These covariates were selected on the basis of literature about PHLF and its risk factors. The variance inflation factors (VIFs) for covariates were clearly below the threshold of ten, which indicated the lack of strong multicollinearity in the models. We also performed sensitivity analyses to test the robustness of the multivariate results. In sensitivity analyses, we only adjusted gender, operation time, and hemorrhage according to the univariate comparing between PHLF and non-PHLF and univariate regression (*P* < 0.100), considering the sample size in this study. In GLMMs, research center was incorporated as a random effect, and the covariates were incorporated as fixed effects. The effect estimates were presented as odds ratios (ORs) for PHLF per an IQR increase in liver volume variables, with corresponding 95% confidence intervals (CIs). Moreover, the values of variables for predicting PHLF were assessed using receiver operating characteristics (ROC) curve analysis, and the area under the curve (AUC) was calculated to compare among these liver volume variables. The critical point of cutoff value was determined by finding the point closest to the upper left corner on the ROC curve.

GLMMs were performed using SAS version 9.4 (SAS Institute Inc., Cary, NC), and other statistical analysis was performed using R studio software (R version 4.3.2). The visualization of the results was carried out in GraphPad Prism version 8 (https://www.graphpad.com). A *P*-value < 0.05 was considered statistically significant for a two-tailed test.

## Results

### Patient Characteristics

**Fig. 1:**
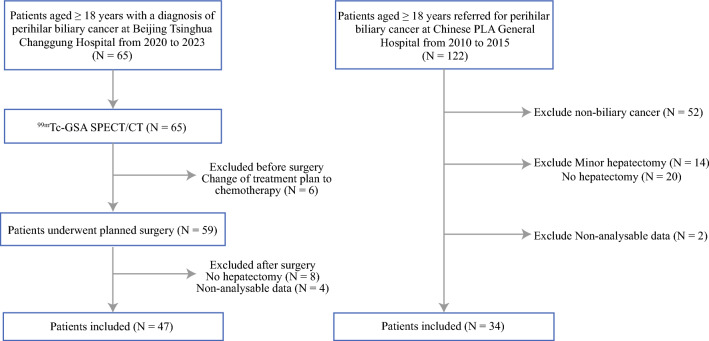
Flowchart of patient selection

During the study period, a total of 187 patients underwent ^99m^Tc-GSA SPECT/CT for curative intent in two hospitals. Of these, a total of 81 patients were included after the exclusion of 52 with nonbiliary cancer disease, of 42 patients without major liver resection, of 6 patients because SPECT/CT were not analyzable, and 6 patients for changing treatment plan. As a result, the remaining 81 patients formed the study cohort (Fig. 1). The median age of patients was 60 years (range, 30–79 years), and 52 (64.20%) patients were men. Most of the enrolled patients had obstructive jaundice requiring preoperative management through PTBD (80.20%). PVE was successfully completed without complication in 28 patients for insufficient FLR followed with liver resection. The average waiting time between PVE and hepatectomy was 36 days. Embolic materials were mainly steel coil together with gelfoam, lipiodol, or polyvinyl alcohol (PVA) consistent with former research.^27^ There were 17 right portal vein embolism cases, 9 right anterior and left portal vein branch embolism cases, and 2 left branch embolism cases. A total of two patients underwent HVE owing to inadequate hypertrophy after PVE. The median preoperative ALT and TBil were 54.9 U/L and 53.3 μmol/L, respectively.

According to ISGLS classification, 22 (27.16%) patients were found to have PHLF; grade B or C PHLF occurred in 14 patients (17.28%). Overall, the main pathological diagnosis was perihilar cholangiocarcinoma, accounting for 88.9% of all patients. Baseline characteristics and pathological diagnosis of patients are summarized in Table 1. Patients with PHLF were older (mean ± SD 61.77 ± 9.57 years versus 59.41 ± 9.64 years, *P* = 0.328), but without statistical significance, and had a higher proportion of male subjects (81.82% versus 57.63%, *P* = 0.043). Liver biochemical tests before surgery showed no significant difference in alanine aminotransferase, total bilirubin, international normalized ratio, or albumin between PHLF and non-PHLF groups (Table 2). The proportion of patients completing PTBD (81.82% versus 79.66%, *P* = 1.000) and PVE (40.91% versus 32.20%, *P* = 0.464) in the two groups were similar. Several ICG-related indicators indices, such as ICGK, ICGK-F, and ICGR15, showed no significant differences between patients with and without PHLF (Table 2). Patients with and without PHLF had comparable MLV of FLR (median 786.13 ml versus 666.00 ml, *P* = 0.066). For the morphological volume related indictors, the patients with PHLF showed lower MLV_FLR_/SLV and higher resection rate than the patients without PHLF (median 0.52 versus 0.67 for MLV_FLR_/SLV, *P* = 0.028; median 0.57 versus 0.46 for resection rate, *P* = 0.002). The ^99m^Tc-GSA indicators of FLR regional liver in two groups were evaluated (Table 2). Patients without PHLF showed higher preoperative FLV_FLR_ (median 440.59 ml versus 335.71 ml, *P* = 0.022), FLV_FLR_/FLV_TOTAL_ (median 0.69 versus 0.52, *P* = 0.013), GSAK_FLR_ (median 0.17/s versus 0.14/s, *P* = 0.137), FLV_FLR_–BWR (median 0.67% versus 0.55%, *P* = 0.005), and PRI (median 0.50 versus 0.41, *P* = 0.023) than patients with PHLF.

**Table 1:** Baseline characteristics of patients (n = 81)

Characteristics (*n* = 81)	Value
Men:women, *n*	52:29
Age (years), median (range)	60 (30–79)
Height (cm), median (range)	166 (148–185)
Weight (kg), median (range)	65 (40–98)
Preoperative biliary drainage, *n* (%)	65 (80.2)
PVE, *n* (%)	28 (34.6)
Preoperative ALT (U/L), median (range)	54.9 (14.0–404.4)
Preoperative TBil (μmol/L), median (range)	53.3 (4.3–610.0)
Preoperative Alb (g/L), median (range)	37.1 (28.9–45.7)
Preoperative INR, median (range)	1.02 (0.80–1.26)
PHLF Grade B/C, *n* (%)	14 (17.3)
Pathological diagnosis, *n* (%)	
Perihilar cholangiocarcinoma	72 (88.9)
Gallbladder adenocarcinoma infiltrating perihilar bile duct	3 (3.7)
Intraductal papillary carcinoma	2 (2.5)
Other	4 (4.9)

The other four cases of pathological diagnoses include leiomyosarcoma, mucinous cystic carcinoma, combined hepatocellular-cholangiocarcinoma, and metastatic adenocarcinoma separately

*PVE* portal vein embolization, *ALT* alanine aminotransferase, *TBil* total bilirubin, *INR* international normalized ratio, *Alb* albumin, *PHLF* post-hepatectomy liver failure

**Table 2:** Characteristics of patients with PHLF and non-PHLF

Variables	Total	Status of PHLF		*P-*values
		No PHLF	PHLF	
	*N* = 81	*N* = 59	*N* = 22	
*Demographic data*				
Age, mean ± SD, years	60.05 ± 9.62	59.41 ± 9.64	61.77 ± 9.57	0.328
Gender, *n* (%)				0.043*
Male	52 (64.20)	34 (57.63)	18 (81.82)	
Female	29 (35.80)	25 (42.37)	4 (18.18)	
BMI, mean ± SD, kg/m^2^	23.46 ± 2.95	23.49 ± 3.06	23.38 ± 2.70	0.877
*Preoperative data*				
ALT, median (IQR), U/L	54.90 (41.20)	52.10 (45.00)	55.80 (29.60)	0.342
TBil, median (IQR), μmol/L	53.30 (75.70)	53.68 (60.60)	50.75 (108.10)	0.698
ALB, median (IQR), g/L	37.10 (5.30)	37.70 (5.60)	36.55 (3.20)	0.356
INR, median (IQR)	1.02 (0.15)	1.02 (0.16)	1.02 (0.15)	0.545
PTBD, *n* (%)	65 (80.25)	47 (79.66)	18 (81.82)	1.000
PVE, *n* (%)	28 (34.57)	19 (32.20)	9 (40.91)	0.464
MLV_FLR_, median (IQR), ml	745.86 (382.02)	786.13 (420.89)	666.00 (179.75)	0.066
MLV_FLR_/SLV, median (IQR)	0.61 (0.31)	0.67 (0.34)	0.52 (0.15)	0.028*
Resection rate, median (IQR)	0.51 (0.22)	0.46 (0.23)	0.57 (0.08)	0.002*
ICGK, median (IQR), /min	0.07 (0.04)	0.07 (0.04)	0.06 (0.03)	0.249
ICGK-F, median (IQR)	0.07 (0.04)	0.07 (0.04)	0.06 (0.03)	0.255
ICGR15, median (IQR), %	8.50 (7.80)	8.35 (6.60)	9.10 (7.90)	0.555
FLV_FLR_, median (IQR), ml	415.30 (273.98)	440.59 (322.05)	335.71 (174.03)	0.022*
FLV_FLR_/FLV_TOTAL_, median (IQR)	0.66 (0.26)	0.69 (0.26)	0.52 (0.24)	0.013*
FLV_FLR_/MLV_FLR_, median (IQR)	0.55 (0.23)	0.56 (0.27)	0.53 (0.16)	0.136
GSAK_FLR_, median (IQR), /s	0.17 (0.10)	0.17 (0.09)	0.14 (0.14)	0.137
FLV_FLR_–BWR, median (IQR), %	0.63 (0.35)	0.67 (0.54)	0.55 (0.18)	0.005*
PRI, median (IQR)	0.46 (0.32)	0.50 (0.38)	0.41 (0.22)	0.023*
*Operative parameters*				
Operation time, median (IQR), min	630.00 (193.00)	625.00 (226.00)	636.50 (302.00)	0.247
Liver resection type, *n* (%)				0.267
Right hemihepatectomy	39 (48.15)	27 (45.76)	12 (54.54)	
Extended right hemihepatectomy	2 (2.47)	1 (1.69)	1 (4.55)	
Right trisectionectomy	4 (4.94)	3 (5.09)	1 (4.55)	
Left hemihepatectomy	27 (33.33)	23 (38.98)	4 (18.18)	
Left trisectionectomy	8 (9.88)	4 (6.78)	4 (18.18)	
Other	1 (1.23)	1 (1.70)	0 (0.0)	
Vascular reconstruction*, *n* (%)	26 (32.10)	18 (30.51)	8 (36.36)	0.616
Hemorrhage, median (IQR), mL	420.00 (500.00)	400.00 (400.00)	615.00 (580.00)	0.002*
Transfusion, median (IQR), mL	0.00 (720.00)	0.00 (435.00)	600.00 (800.00)	0.044*
*Postoperative outcomes*				
PHLF grade,* n* (%)				NA
No	59 (72.84)	59 (100.00)	0 (0.00)	
A	8 (9.88)	0 (0.00)	8 (36.36)	
B	9 (11.11)	0 (0.00)	9 (40.91)	
C	5 (6.17)	0 (0.00)	5 (22.73)	
50-50 Criteria, *n* (%)	24 (29.63)	11 (18.64)	13 (59.09)	< 0.001*
Abdominal infectious, *n* (%)	20 (24.69)	12 (20.34)	8 (36.36)	0.137

^*^, Vascular reconstruction included hepatic artery/portal vein reconstruction or both; *50-50 Criteria* the conjunction of prothrombin time < 50% and serum bilirubin > 50 μmol/L on the fifth postoperative day; *PHLF* post-hepatectomy liver failure, *BMI* body mass index, *PVE* portal vein embolization, *ALT* alanine aminotransferase, *TBil* total bilirubin, *INR* international normalized ratio, *Alb* albumin, *PTBD* percutaneous transhepatic biliary drainage, *MLV*_*FLR*_ morphological liver volume of future liver remnant, *SLV* standard liver volume, *ICGK* the clearance rate of indocyanine green, *ICGK-F* the clearance rate of indocyanine green of future liver remnant, *ICGR15* the indocyanine green retention rate at 15 min, *FLV*_*FLR*_ functional liver volume of future liver remnant, *FLV*_*TOTAL*_ functional liver volume of total liver, *GSAK*_*FLR*_ the GSA disappearance rate constant of future liver remnant, *FLV*_*FLR*_*–BWR* ratio of the functional volume of future liver remnant to body weight, *PRI* predictive residual index

For the intraoperative parameters, the patients with PHLF had comparable operation times (median 636.50 min versus 625.00 min, *P* = 0.247) and comparable liver resection type (*P* = 0.267), but higher intraoperative blood loss (median 615.00 ml versus 400.00 ml, *P* = 0.002) and intraoperative blood transfusion (median 600.00 ml versus 0.00 ml, *P* = 0.044) than the patients without PHLF. As for the postoperative outcomes, the proportion of 50-50 Criteria-positive patients was significantly higher in the PHLF group (59.09% versus 18.64%, *P* < 0.001), while postoperative abdominal infections showed no difference between the two groups (36.36% versus 20.34%, *P* = 0.137).

### Regional Liver Function of Embolized and Non-embolized Lobes in PVE

**Fig. 2:**
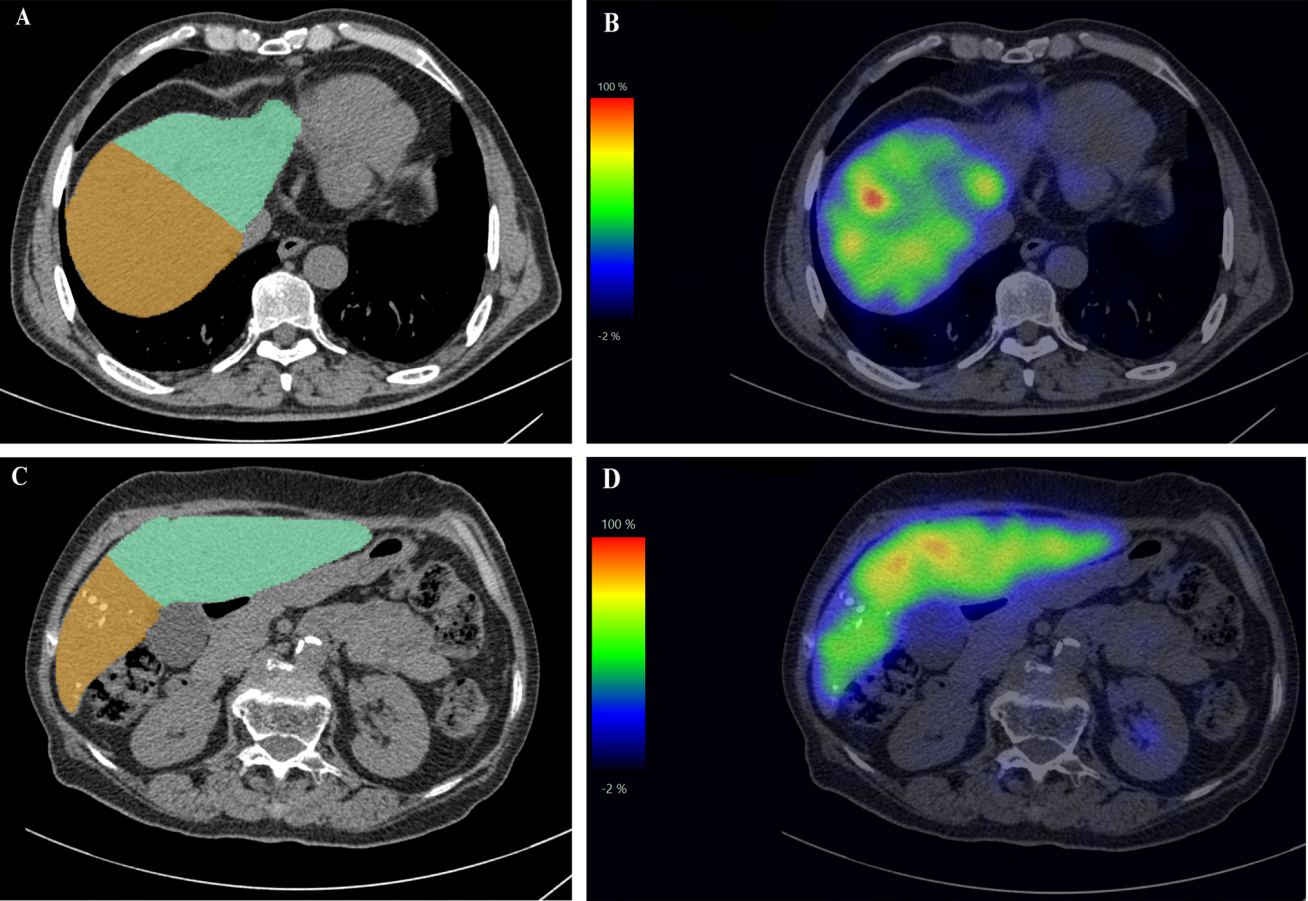
^99m^Tc-GSA SPECT/CT fusing image and CT images of two patients before hepatectomy; CT map (**A**) and ^99m^Tc-GSA SPECT/CT fusing map (**B**) of a male patient (age 66) with perihilar cholangiocarcinoma (pCCA) who underwent left hemihepatectomy and the green area is resection liver region; CT map (**C**) and ^99m^Tc-GSA SPECT/CT fusing map (**D**) of another female patient (age 74 years) with pCCA who underwent right hemihepatectomy after portal vein embolization (PVE) and the green area is the remnant liver region

Figure 2 shows examples of ^99m^Tc-GSA SPECT/CT fusing maps for a patient without PVE (man, age 66 years) and a patient with PVE (woman, age 74 years). Inhomogeneous liver function distribution can be observed in the whole livers and in the remnant regions of both patients. A total of 28 patients were treated with PVE before hepatectomy, and the regional liver function of embolized and nonembolized lobes were compared (Supplementary Fig 1). The median MLV of embolized side was higher than the nonembolized side (*P* = 0.001), while the median FLV (*P* = 0.056), FLV/MLV (*P* <0.0001), FLV-BWR (*P* = 0.047), and GSAK (*P* = 0.136) of nonembolized lobes were higher than embolized lobes.

### Association between FLR Function and PHLF

Table 3 summarizes the results of the univariable analysis. Demographic and preoperative liver biochemical indicators were not associated with PHLF. Univariable analyses showed that MLV_FLR_/SLV and resection rate were related to the probability of PHLF (Table 3, *P* < 0.05). The FLV_FLR_, FLV_FLR_/FLV_TOTAL_, FLV_FLR_–BWR, and PRI were inversely related to the probability of PHLF (Table 3, *P* < 0.05 for all parameters). To avoid multicollinearity, multivariate analyses of generalized linear mixed models (GLMMs) adjusting for age, gender, BMI, ALT, TBil, ALB, INR, PTBD, PVE, operation time, and hemorrhage were applied to each of the preoperative liver function parameters, respectively. The results indicated that a decrease of MLV_FLR_/SLV (*P* = 0.018), FLV_FLR_ (*P* = 0.004), FLV_FLR_/FLV_TOTAL_ (*P* = 0.050), FLV_FLR_–BWR (*P* = 0.003), or PRI (*P* = 0.009), or an increased resection rate (*P* = 0.016), were independently associated with a higher probability of PHLF, respectively (Table 4). In sensitivity analyses, we only adjusted gender, operation time, and hemorrhage according to univariate results. All estimates were stable (Supplementary Fig 2).

**Table 3:** Univariate analyses between factors and PHLF

Variables	OR	95% CI		*P*-value
		Lower	Upper	
*Demographic data*				
Age (per 1 year)	1.027	0.974	1.083	0.328
Gender, male	3.309	0.996	10.987	0.301
BMI (per 1 kg/m2)	0.987	0.835	1.166	0.876
*Preoperative data*				
ALT, U/L, per IQR	0.902	0.628	1.297	0.579
TBil, μmol/L, per IQR	1.116	0.799	1.558	0.523
ALB, g/L, per IQR	0.729	0.355	1.496	0.391
INR, per IQR	1.285	0.640	2.580	0.482
PTBD	1.149	0.327	4.031	0.864
PVE	1.457	0.531	4.002	0.824
MLV_FLR_, ml, per IQR	0.480	0.210	1.097	0.086
MLV_FLR_/SLV, per IQR	0.330	0.129	0.843	0.023*
Resection rate, per IQR	4.197	1.592	11.069	0.005*
ICGK, /min, per IQR	0.482	0.169	1.376	0.180
ICGR15, %, per IQR	1.171	0.695	1.974	0.555
ICGK-F, per IQR	0.482	0.169	1.376	0.180
FLV_FLR_, ml, per IQR	0.228	0.082	0.636	0.006*
FLV_FLR_/FLV_TOTAL_, per IQR	0.413	0.199	0.859	0.020*
GSAK_FLR_, /s, per IQR	0.561	0.240	1.308	0.185
FLV_FLR_–BWR, %, per IQR	0.272	0.118	0.627	0.003*
PRI, per IQR	0.193	0.065	0.578	0.004*
*Operative data*				
Operation time, min, per IQR	1.634	0.923	2.892	0.096
Liver resection type	–	–	–	–
Vascular reconstruction*	0.704	1.621	4.557	0.500
Hemorrhage, ml, per IQR	1.648	1.046	2.597	0.034*
Transfusion, ml, per IQR	1.837	1.030	3.277	0.043*

^*^, Vascular reconstruction included hepatic artery/portal vein reconstruction or both

*PHLF* post-hepatectomy liver failure, *BMI* body mass index, *PVE* portal vein embolization, *ALT* alanine aminotransferase, *TBil* total bilirubin, *INR* international normalized ratio, *Alb* albumin, *PTBD* percutaneous transhepatic biliary drainage, *MLV*_*FLR*_ morphological liver volume of future liver remnant, *SLV* standard liver volume, *ICGK* the clearance rate of indocyanine green, *ICGK-F* the clearance rate of indocyanine green of future liver remnant, *ICGR15* the indocyanine green retention rate at 15 min, *FLV*_*FLR*_ functional liver volume of future liver remnant, *FLV*_*TOTAL*_ functional liver volume of total liver, *GSAK*_*FLR*_ the GSA disappearance rate constant of future liver remnant, *FLV*_*FLR*_*–BWR* ratio of the functional volume of future liver remnant to body weight, *PRI* predictive residual index

**Table 4:** Multivariate analyses between factors and PHLF by generalized linear mixed models (GLMMs)

Variables	Crude models^a^				Adjusted models^b^			
	OR	95% CI		*P*-value	OR	95% CI		*P*-value
MLV_FLR_, ml, per IQR	0.480	0.210	1.097	0.086				
MLV_FLR_/SLV, per IQR	0.330	0.129	0.843	0.023*	0.185	0.047	0.723	0.018*
Resection rate, per IQR	4.197	1.592	11.069	0.005*	5.283	1.412	19.762	0.016*
ICGK, /min, per IQR	0.482	0.169	1.376	0.180				
ICGR15, %, per IQR	1.171	0.695	1.974	0.555				
ICGK-F, per IQR	0.482	0.169	1.376	0.180				
FLV_FLR_, ml, per IQR	0.228	0.082	0.636	0.006*	0.114	0.028	0.465	0.004*
FLV_FLR_/FLV_TOTAL_, per IQR	0.413	0.199	0.859	0.020*	0.426	0.188	0.969	0.050*
FLV_FLR_–BWR, %, per IQR	0.272	0.118	0.627	0.003*	0.170	0.055	0.526	0.003*
PRI, per IQR	0.193	0.065	0.578	0.004*	0.163	0.044	0.609	0.009*
Hemorrhage, ml, per IQR	1.648	1.046	2.597	0.034*	1.676	0.955	2.942	0.076
Transfusion, ml, per IQR	1.837	1.030	3.277	0.043*	1.822	0.865	3.838	0.119

^a^GLMMs were not adjusted for other factors

^b^GLMMs were adjusted for age, gender, BMI, ALT, TBil, ALB, INR, PTBD, PVE, operation time, and hemorrhage

*PHLF* post-hepatectomy liver failure, *MLV*_*FLR*_ morphological liver volume of future liver remnant, *SLV* standard liver volume, *ICGK* the clearance rate of indocyanine green, *ICGK-F* the clearance rate of indocyanine green of future liver remnant, *ICGR15* the indocyanine green retention rate at 15 min, *FLV*_*FLR*_ functional liver volume of future liver remnant, *FLV*_*TOTAL*_ functional liver volume of total liver, *FLV*_*FLR*_*–BWR* ratio of the functional volume of future liver remnant to body weight, *PRI* predictive residual index

The AUCs with 95% CI of these preoperative parameters are listed in Table 5. In crude model, FLV_FLR_–BWR showed a relatively better AUC than other different predictors for early warning PHLF. The AUCs of FLV_FLR_, FLV_FLR_–BWR and PRI, which showed a better prediction ability of PHLF among indicators, were larger than that of MLV_FLR_, MLV_FLR_/SLV, ICGK, ICGR15, and ICGK-F in adjusted GLMMs models. The ROC curves of different preoperative liver function parameters are shown in Supplementary Fig 3. According to the crude model, the best FLV_FLR_–BWR cutoff value was 0.82%, with a sensitivity of 100% and a specificity of 40.7%, to distinguish patients from those without PHLF. The positive predictive value (PPV) and negative predictive value (NPV) of this cutoff value were 38.6% and 100%.

**Table 5:** ROCs of factors with PHLF by generalized linear mixed models (GLMMs)

Variables	Crude models^a^					Adjusted models^b^				
	AUC	95% CI		Sensitivity	Specificity	AUC	95% CI		Sensitivity	Specificity
MLV_FLR_, ml	0.636	0.506	0.766	0.905	0.441	0.789	0.689	0.890	1.000	0.508
MLV_FLR_/SLV	0.663	0.541	0.785	0.864	0.492	0.799	0.702	0.896	1.000	0.525
ICGK, /min	0.637	0.461	0.813	0.615	0.647	0.765	0.619	0.911	1.000	0.471
ICGK-F	0.614	0.435	0.794	0.615	0.647	0.760	0.613	0.907	1.000	0.471
ICGR15, %	0.558	0.362	0.754	0.385	0.794	0.765	0.618	0.911	1.000	0.471
FLV_FLR_, ml	0.666	0.549	0.784	0.909	0.475	0.830	0.738	0.922	0.955	0.576
FLV_FLR_–BWR, %	0.704	0.592	0.817	1.000	0.407	0.835	0.746	0.925	1.000	0.559
PRI	0.668	0.546	0.790	0.909	0.407	0.802	0.705	0.899	1.000	0.559

^a^GLMMs were not adjusted for other factors

^b^GLMMs were adjusted for age, gender, BMI, ALT, TBil, ALB, INR, PTBD, PVE, operation time, and hemorrhage

*MLV*_*FLR*_ morphological liver volume of future liver remnant, *SLV* standard liver volume, *ICGK* the clearance rate of indocyanine green, *ICGK-F* the clearance rate of indocyanine green of future liver remnant, *ICGR15* the indocyanine green retention rate at 15 min, *FLV*_*FLR*_ functional liver volume of future liver remnant, *FLV*_*FLR*_*–BWR* ratio of the functional volume of future liver remnant to body weight, *PRI* predictive residual index

## Discussion

Malignant perihilar biliary tumors often necessitate extensive liver resection for curative treatment. The mortality rate following extended hepatic resection is relatively high in biliary tract malignancies.^2,31^ The PHLF is a major contributor to high mortality and morbidity rate. Therefore, investigating factors that impact PHLF is of great interest for surgeons aiming to enhance postoperative outcomes in patients with biliary tract malignancies. Possible causes of PHLF could be cholangitis and complex surgeries such as left trisectionectomy,^32^ and numerous studies have highlighted insufficient FLR as a key factor in biliary tract malignancies or pCCA.^31,33–35^ Moreover, the availability of preoperative PVE has further increased the difficulty of the preoperative assessment of regional hepatic function owing to functional heterogeneity among different lobes.^18^ Accurate assessment of regional liver function before liver resection is crucial for short-term prognosis preventing PHLF, particularly in patients with obstructive jaundice necessitating major liver resection. In this study, we identified FLV-BWR as the most useful independent parameter for the prediction of ISGLS-PHLF in cholestasis by real-world data from two centers.

In surgical planning, decision trees are commonly used to integrate parameters that reflect overall liver function, such as total bilirubin levels, Child–Pugh scores, and ICGR15, along with the FLR volume ratio to identify high-risk patients. However, the presence of heterogeneous liver function distribution as discussed in patients with hepatic disease, coupled with the impact of jaundice and PVE on ICG and CT results, can render the results unreliable. None of these criteria directly quantify this uneven functional distribution, thereby affecting their accuracy. Nuclear medicine imaging or functional magnetic resonance imaging are the main methods used for evaluating regional liver function currently. Gadolinium ethoxybenzyl-diethylenetriaminepentaacetic acid (Gd-EOB-DTPA)-enhanced MRI is valuable not only for detecting liver tumors, but also for evaluating regional liver function and predicting PHLF.^36^ However, partial excretion of Gd-EOB-DTPA through the biliary tract restricts its use in biliary tumors with obstructive jaundice. ^99m^Tc-GSA SPECT/CT and ^99m^Tc-mebrofenin hepatobiliary scintigraphy (HBS) are also widely used to assess regional liver function.^11,12,14^ The ^99m^Tc-GSA SPECT/CT was proven to be more accurate than ICG in cases of ICG excretory defects or jaundice, where ICG values were extremely deteriorated.^37^ Many scholars have used ^99m^Tc-GSA SPECT/CT to develop indicators for predicting short-term postoperative outcomes such as PHLF, morbidity, or mortality.^11,12^ However, these are retrospective studies with small sample sizes in a single center. Sumiyoshi et al. retrospectively enrolled 30 patients, proving the accuracy and suitability of remnant KGSA in pCCA without ROC analysis to determine the cutoff value of remnant KGSA.^16^ Okabayashi et al. found that remnant KGSA ≥ 0.05 enabled postoperative safety in single center recruiting a small number of patients with mass-forming intrahepatic cholangiocarcinoma.^19^ There is no multicenter evidence supporting the superiority of nuclear imaging over conventional methods in cholestasis liver. This is the first dual-center cohort study to provide novel findings on the use of ^99m^Tc-GSA SPECT/CT in identifying patients with high-risk malignant perihilar biliary tumor with PHLF. The study also demonstrates that ICG and CT have limited capability for differentiating patients with PHLF from those with sufficient FLR liver function, including patients who have undergone PVE.

An ICGK-F of 0.05 is a widely used threshold in Japan for biliary cancer before liver resection derived from large-scale retrospective cohort studies,^20,26,27^ which can significantly reduce postoperative mortality rate. When bilirubin levels are high, the ICG test results should be interpreted with caution.^38^ The uptake of ^99m^Tc-GSA by hepatocyte via ASGPR can be used to obtain inhomogeneous regional liver function by SPECT/CT, and it is not directly inhibited by hyperbilirubinemia. Satoh et al. proposed a refined technique for calculating functional liver volume on the basis of the level of ^99m^Tc-GSA radioactivity in each voxel.^14,24^ Through this method, our previous research demonstrated that PRI is a good indicator for liver function on different drainage sides of liver lobes.^21^ In the present study, we observed that the median values of FLV, FLV/MLV, FLV-BWR, and GSAK were higher in the nonembolized lobe compared with the embolized lobe. This finding suggests that the functional capacity of the nonembolized side after PVE was greater than that of the embolized side, despite the larger MLV of the embolized side. To compare the increasing ratio of FLR in patients with PVE, Beppu et al. discovered that after a total of 3–5 weeks of PVE, the hepatic functional proliferation exceeded the volumetric reaction, indicating the potential of ^99m^Tc-GSA SPECT/CT as a monitoring tool for assessing the efficacy of PVE.^39^ However, more data before PVE should have been collected to prove it in our study. The FLV, the ratio of FLV, FLV–BWR, and PRI indexes of regional liver, were significantly lower in the PHLF than the non-PHLF group, which confirmed the superiority of ^99m^Tc-GSA test. The larger AUCs of FLV_FLR_–BWR and FLV_FLR_ also demonstrated the advantages of ^99m^Tc-GSA SPECT/CT compared with conventional ICG parameters.

Body weight and morphological volume are commonly used indicators to assess graft suitability before liver transplantation to prevent small-for-size syndrome. The appropriateness of the graft volume in relation to the recipient’s weight is typically assessed via the graft-to-body weight ratio, with the minimum acceptable graft volume being approximately 0.8% of the recipient’s body weight.^40^ However, in urgent transplant scenarios such as fulminant hepatic failure, successful transplantation has been achieved using small-for-size grafts constituting just 0.6% of the recipient’s body weight.^40^ Some scholars have applied this concept for FLR assessment before hepatectomy in pCCA.^25^ Hayashi et al. found that the liver-residual-volume-to-body-weight ratio of 0.65% could be utilized to select patients before right trisectionectomy, demonstrating the superiority of this indicator over ICGK-F^41^. However, unlike previous studies^25,40–42^ that used a combination of body weight and morphological volume of different liver segments to evaluate the liver function, this study used functional volume and body weight to quantify the pixelwise remnant liver function and liver volume. This provided a more accurate and practical method for clinical use. On the basis of the calculation method, FLV represents the liver tissue volume weighted by the relative amount of ^99m^Tc-GSA uptake, which is proportional to the number of ASGPR. Therefore, the magnitude of FLV is related to both the liver tissue volume and the amount of ASGPR on liver cells. To a certain extent, FLV reflects the relative total number of ASGPR in the liver. FLV_FLR_–BWR does not assume uniformly preserved liver function and emphasizes that body weight reflects the minimal metabolic demands of patients.^25,42^ Univariate and multivariate analyses found that FLV–BWR was an independent predictive factor of PHLF in our study, with a larger AUC compared with other ICG and CT parameters when considering preoperative and intraoperative indicators comprehensively in GLMMs. Several studies analyzed the characteristics of the cutoff values in predicting postoperative PHLF. However, they were mainly results from hepatocellular carcinoma or colorectal liver metastasis.^12^ In general, the sensitivity and specificity of the cutoff values varied considerably, from 50 to 100% and from 32 to 96%, respectively. The PPV of the cutoffs varied considerably as well (7–92%), whereas the NPV were consistently high (82–100%).^11^ In the current study, with a cutoff of 0.82%, the sensitivity and specificity of FLV_FLR_–BWR in distinguishing PHLF were 100% and 40.7%, respectively.

HBS has emerged as another increasingly utilized clinical tool for preoperative risk assessment in patients undergoing major liver resection. The guideline recommends that HBS may provide clinical benefits for patients scheduled for major liver resection with serum bilirubin levels < 2.92 mg/dL, particularly in cases showing heterogeneous liver function distribution.^43^ Olthof et al. established a threshold of 8.5%/min for patients with suspected pCCA. Application of this functional cutoff value demonstrated a NPV of 94% in the subgroup of patients with bilirubin levels below 2.92 mg/dL.^44^ Their findings indicate that HBS can also be used to evaluate FLR function in patients with pCCA as a validated alternative method when bilirubin levels remain within low ranges. However, the currently available data are insufficient to establish advantages or disadvantages over different nuclear imaging tracers such as ^99m^Tc-GSA and ^99m^Tc-mebrofenin.^12^

In our study, the preoperative GSAK of FLR in the non-PHLF group was higher than that of the PHLF group, indicating the feasibility of GSAK in evaluating regional liver function in patients even after PVE. However, more data may be necessary because it was not significant in univariate and multivariate analyses. Hemorrhage and transfusion were statistically different between PHLF and non-PHLF groups, but they displayed no significance in multivariate analysis. These results align with a study that found adjusted blood loss should be less than 10 mL/kg to minimize postoperative complications.^45^ Some scholars reported that operation time, extent of hepatectomy, blood loss volume, and aspartate aminotransferase to platelet ratio index had significant predictive value for predicting liver failure.^12^

The current study has several limitations. First, patients with poor liver function recruited in this study did not undergo liver resection surgery for perioperative safety. More research is needed to determine the GSA cutoff value for this population. Second, the period between ^99m^Tc-GSA SPECT/CT and hepatectomy in a few patients was longer than the average, and the parameters may not represent the actual liver volume and function at the time of hepatectomy. However, changes in the patients such as fever and coronavirus disease 2019 (COVID-19) made the timing of the surgery uncontrollable. Third, patients who underwent radiation therapy, chemotherapy or immunotherapy before liver resection were excluded in this study. In the future, it is critical to investigate the feasibility of the proposed remnant liver function evaluation methods in these patients and to explore the indication and functional change before and after PVE or HVE. Last, the sample size of events in this study was limited for multivariate analyses. However, we adjusted three covariates according to univariate results instead of 11 covariates according to the literature in main models. We found that all results were robust. Moreover, the accuracy of FLV_FLR_–BWR should be validated in a larger cohort of patients with other biliary tract diseases including intrahepatic cholangiocarcinoma.

## Supplementary Information

Below is the link to the electronic supplementary material.

Supplementary file1 Different regional liver function of embolized and non-embolized lobes; Ns: no significance; MLV: morphological liver volume; FLV: functional liver volume; FLV–BWR: ratio of the functional volume to body weight; GSAK: the GSA disappearance rate constant. *: P<0.05; **: P<0.01; ****: P<0.0001

Supplementary file2 Associations between FLR function and PHLF; Crude model: GLMMs were not adjusted for other factors; Adjusted model 1 (main model): GLMMs were adjusted for age, gender, BMI, ALT, TBil, ALB, INR, PTBD, PVE, operation time and hemorrhage according to the literature about PHLF and its risk factors; Adjusted model 2 (sensitivity analysis model): GLMMs were adjusted for gender, operation time and hemorrhage according to the univariate results

Supplementary file3 The ROCs of the preoperative parameters in predicting PHLF by generalized linear mixed models (GLMMs); MLV_FLR_: morphological liver volume of future liver remnant; SLV: standard liver volume; ICGK: the clearance rate of indocyanine green; ICGK-F: the clearance rate of indocyanine green of future liver remnant; ICGR15: the indocyanine green retention rate at 15 minutes; FLV_FLR_: functional liver volume of future liver remnant; FLV_FLR_–BWR: ratio of the functional volume of future liver remnant to body weight; PRI: predictive residual index
