# Supplementary figures and images for: Regional Liver Function Assessment Using 99mTc-GSA SPECT/CT Scintigraphy in Malignant Perihilar Biliary Tumor Undergoing Major Liver Resection: A Dual-Center Cohort Study
Source: Ann Surg Oncol. 2025 Apr 17;32(9):6230–42. doi: 10.1245/s10434-025-17207-x (PMC12317906; doi:10.1245/s10434-025-17207-x)

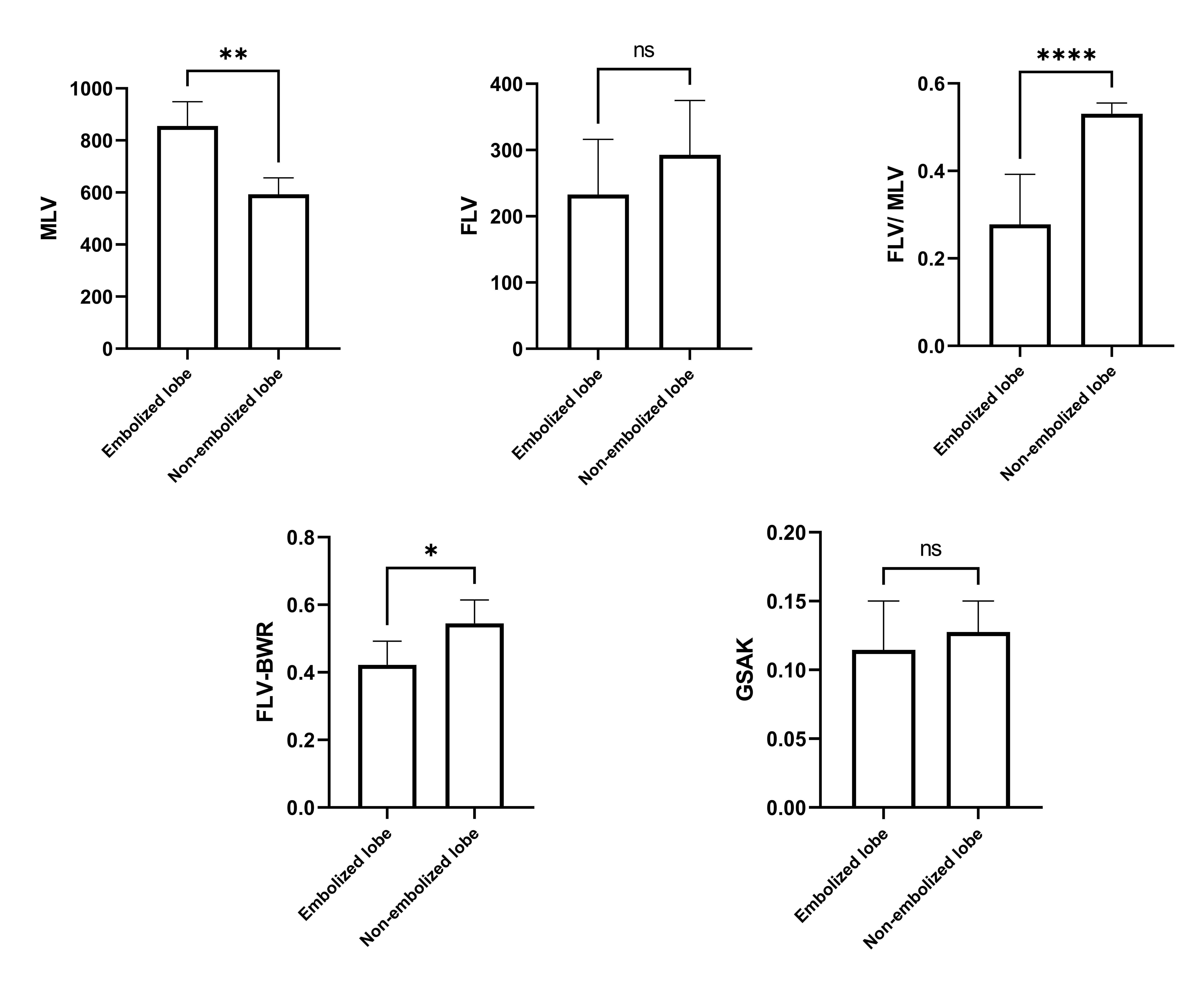

Supplement: Supplementary file 1 — Supplementary file1 Different regional liver function of embolized and non-embolized lobes; Ns: no significance; MLV: morphological liver volume; FLV: functional liver volume; FLV–BWR: ratio of the functional volume to body weight; GSAK: the GSA disappearance rate constant. *: P<0.05; **: P<0.01; ****: P<0.0001 [file 10434_2025_17207_MOESM1_ESM.jpg]

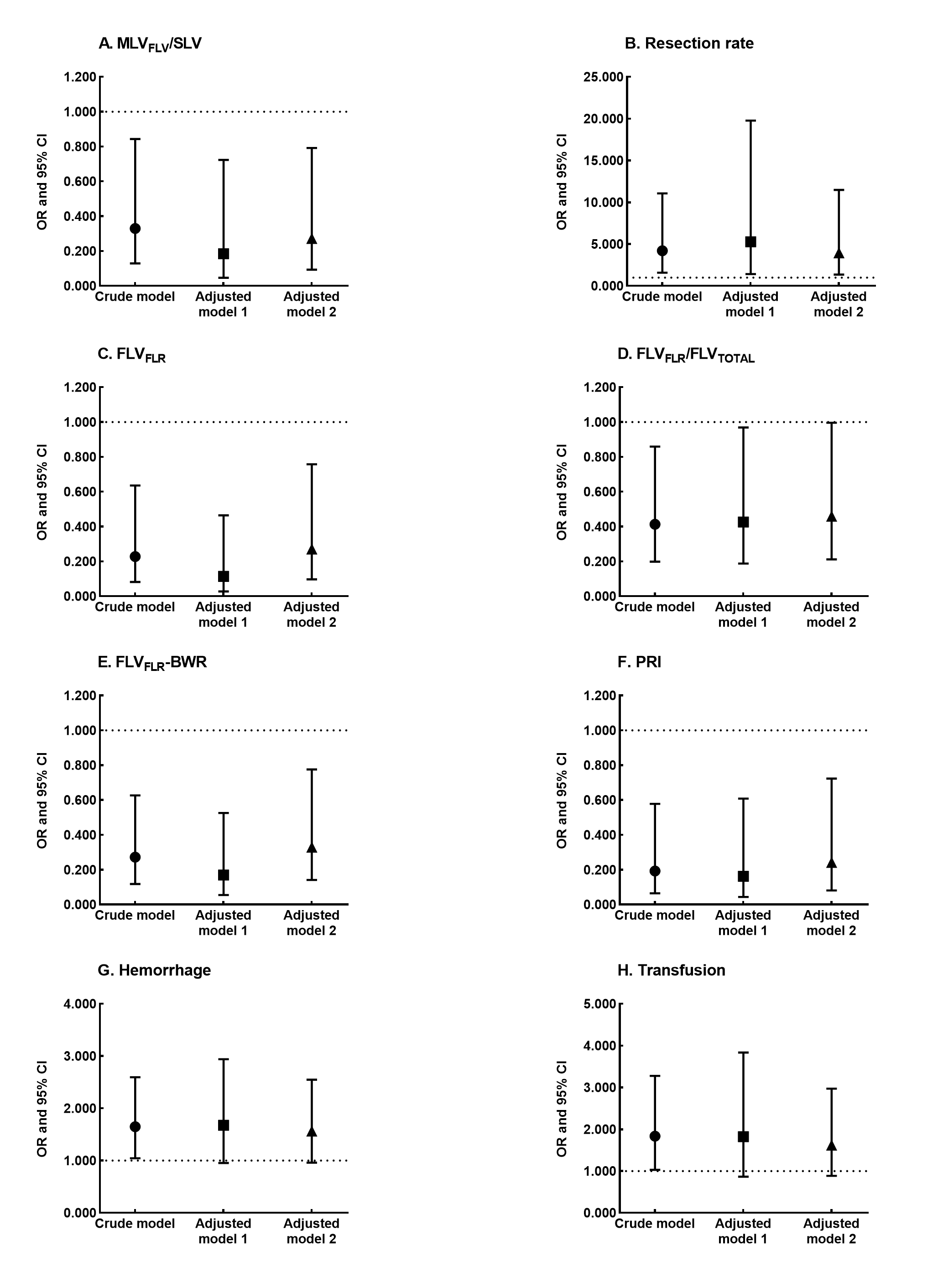

Supplement: Supplementary file 2 — Supplementary file2 Associations between FLR function and PHLF; Crude model: GLMMs were not adjusted for other factors; Adjusted model 1 (main model): GLMMs were adjusted for age, gender, BMI, ALT, TBil, ALB, INR, PTBD, PVE, operation time and hemorrhage according to the literature about PHLF and its risk factors; Adjusted model 2 (sensitivity analysis model): GLMMs were adjusted for gender, operation time and hemorrhage according to the univariate results [file 10434_2025_17207_MOESM2_ESM.tif]

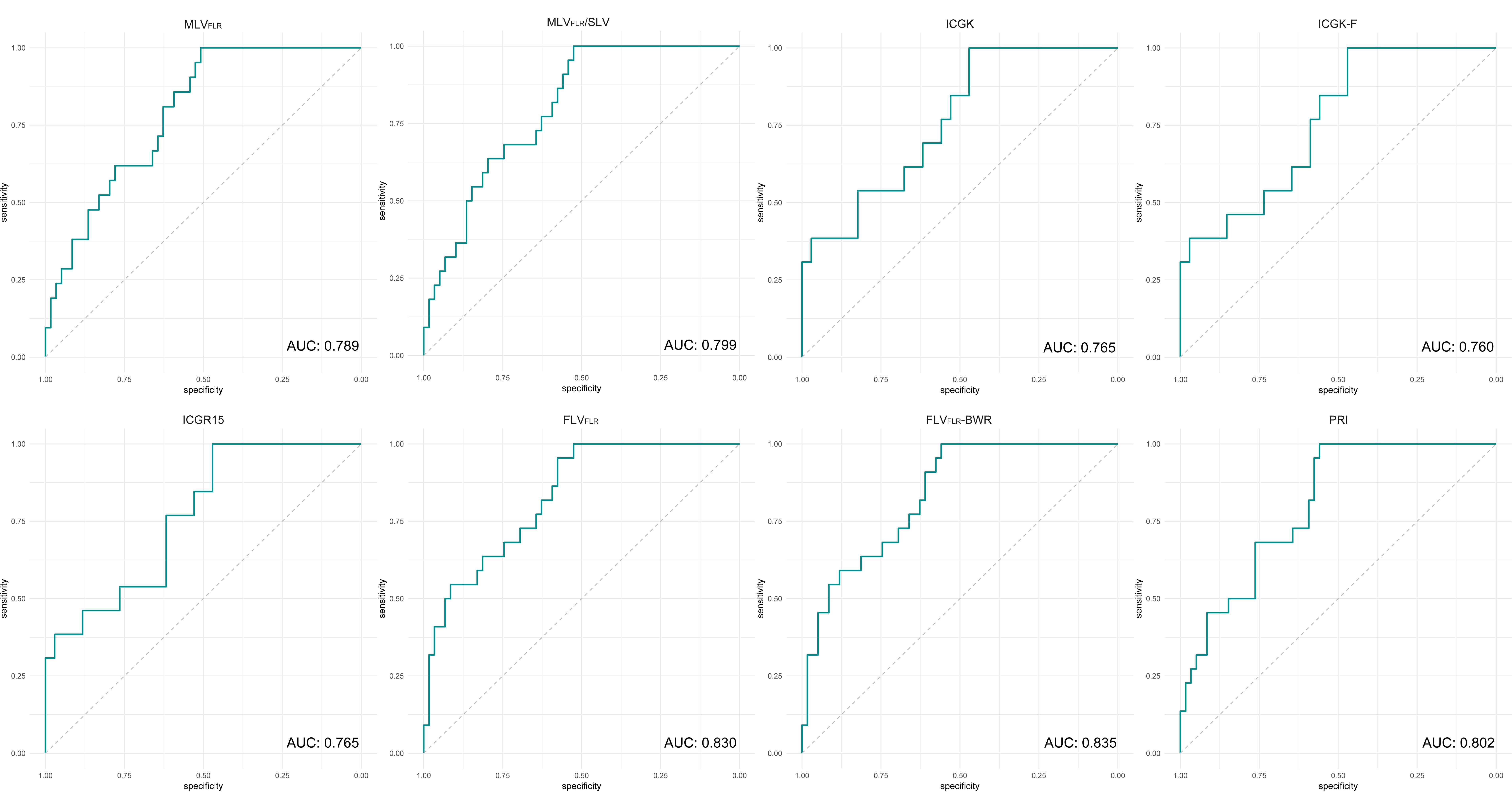

Supplement: Supplementary file 3 — Supplementary file3 The ROCs of the preoperative parameters in predicting PHLF by generalized linear mixed models (GLMMs); MLVFLR: morphological liver volume of future liver remnant; SLV: standard liver volume; ICGK: the clearance rate of indocyanine green; ICGK-F: the clearance rate of indocyanine green of future liver remnant; ICGR15: the indocyanine green retention rate at 15 minutes; FLVFLR: functional liver volume of future liver remnant; FLVFLR–BWR: ratio of the functional volume of future liver remnant to body weight; PRI: predictive residual index [file 10434_2025_17207_MOESM3_ESM.jpg]
